# Endoplasmic reticulum-Golgi intermediate compartment protein 3 knockdown suppresses lung cancer through endoplasmic reticulum stress-induced autophagy

**DOI:** 10.18632/oncotarget.11678

**Published:** 2016-08-29

**Authors:** Seong-Ho Hong, Seung-Hee Chang, Kyung-Cho Cho, Sanghwa Kim, Sungjin Park, Ah Young Lee, Hu-Lin Jiang, Hyeon-Jeong Kim, Somin Lee, Kyeong-Nam Yu, Hwi Won Seo, Chanhee Chae, Kwang Pyo Kim, Jongsun Park, Myung-Haing Cho

**Affiliations:** ^1^ Laboratory of Toxicology, BK21 PLUS Program for Creative Veterinary Science Research, Research Institute for Veterinary Science and College of Veterinary Medicine, Seoul National University, Seoul 08826, Korea; ^2^ New Drug Development Center, Daegu-Gyeongbuk Medical Innovation Foundation, Daegu 41061, Korea; ^3^ Department of Applied Chemistry, College of Applied Science, Kyung Hee University, Yongin 17104, Korea; ^4^ Graduate Group of Tumor Biology, Seoul National University, Seoul 08826, Korea; ^5^ Department of Pharmaceutics, State Key Laboratory of Natural Medicines, China Pharmaceutical University, Nanjing 210009, PR China; ^6^ Laboratory of Pathology, College of Veterinary Medicine, Seoul National University, Seoul 08826, Korea; ^7^ Department of Pharmacology and Medical Science, Infection Signaling Network Research Center, College of Medicine, Chungnam National University, Daejeon 35015, Korea; ^8^ Graduate School of Convergence Science and Technology, Seoul National University, Suwon 16229, Korea; ^9^ Advanced Institute of Convergence Technology, Seoul National University, Suwon 16229, Korea; ^10^ Institute of GreenBio Science Technology, Seoul National University, Pyeongchang-gun 25354, Korea

**Keywords:** lung cancer, gene therapy, endoplasmic reticulum-Golgi intermediate compartment protein 3 (ERGIC3), golgi apparatus, ER stress

## Abstract

Trafficking from the endoplasmic reticulum (ER) to the Golgi apparatus is elevated in cancer cells. Therefore, proteins of the ER-Golgi intermediate compartment (ERGIC) attract significant attention as targets for cancer treatment. Enhanced cancer cell growth and epithelial-mesenchymal transition by ERGICs correlates with poor-prognosis of lung cancer. This prompted us to assess whether knockdown of ERGIC3 may decrease lung cancer growth. To test the hypothesis, the effects of ERGIC3 short hairpin RNA (shERGIC3) on ER stress-induced cell death and lung tumorigenesis were investigated both *in vitro* and *in vivo*. Knockdown of ERGIC3 led to ER stress-induced autophagic cell death and suppression of proliferation in the A549 human lung cancer cell-line. Moreover, non-invasive aerosol-delivery of shERGIC3 using the biocompatible carrier glycerol propoxylate triacrylate and spermine (GPT-SPE) inhibited lung tumorigenesis in the *K-ras^LA1^* murine model of lung cancer. Our data suggest that suppression of ERGIC3 could provide a framework for the development of effective lung cancer therapies.

## INTRODUCTION

The endoplasmic reticulum-Golgi intermediate compartment (ERGIC) is a dynamic and mobile early secretory pathway located between the endoplasmic reticulum (ER) and the Golgi apparatus in mammalian cells [1]. ER-Golgi trafficking is elevated in cancer cells; therefore, ER- and Golgi-related proteins could be good targets for cancer therapy [2].

ERGIC3 is a human ER-related 43-kDa protein (ERp43). Over-expression of ERGIC3 promotes cell growth and significantly reduces ER stress-mediated cell death [3]. ERGIC3 correlates with cell proliferation, migration and epithelial to mesenchymal transition in hepatocellular carcinomas (HCC) [4]. This protein is over-expressed in lung cancer tissues and colorectal tumors [5, 6].

Secretory proteins are properly folded by various enzymes called chaperones, which function in protein folding and promote degradation of misfolded polypeptides [7]. Calcium depletion in the ER lumen, glucose deprivation, inhibition of asparagine (N)-linked glycosylation, and disulfide bond reduction can induce the accumulation of unfolded proteins and disrupt ER function, a phenomenon known as ER stress [8]. Under ER stress conditions, attenuation of translation as well as degradation responses and transcriptional induction of chaperone proteins are activated to prevent unfolded protein accumulation. To increase ER folding capacity, the intracellular signaling pathway known as the unfolded protein response (UPR) is activated, resulting in transcriptional up-regulation of ER resident proteins [9].

ER stress can trigger autophagy, which is considered a downstream mediator in ER stress-induced cell death [10, 11]. Autophagy, known as cellular self-digestion, includes the degradation of cytoplasmic proteins and organelles. Autophagy is activated in cells for protein clearance in conditions such as starvation, irradiation, and hypoxia or in diseases such as cancer [12]. When ER stress or damage cannot be repaired by the UPR pathway, cell death may be induced [13, 14].

ERGIC3 is over-expressed in cancer, and also correlates with oncogenic lung diseases. Moreover, the relation between increased cancer cell growth and epithelial-mesenchymal transition with ERGIC3 led us to investigate the therapeutic effect of ERGIC3 downregulation in lung cancer. In this study, we show that delivery of a small hairpin RNA targeting ERGIC3 (shERGIC3) suppresses lung cancer through ER stress-induced autophagic cell death in both *in vitro* and *in vivo* experiments.

## RESULTS

### Expression of ERGIC3 in human normal lung and lung adenocarcinoma tissues

**Figure 1:**
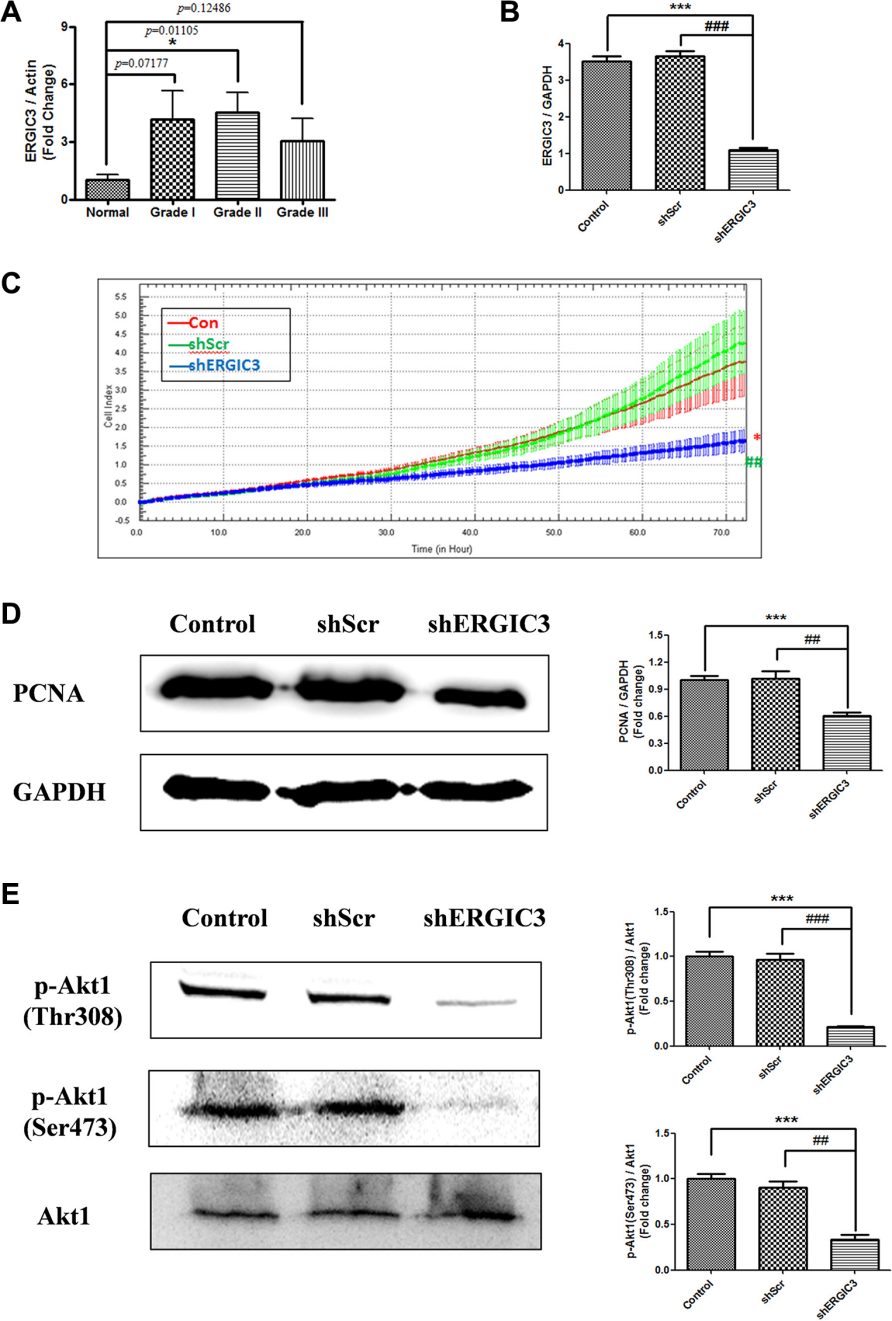
ERGIC3 expression in tumor tissues and effect of shERGIC3 downregulation (**A**) Densitometric analysis of ERGIC3 protein on Western blot in human normal and adenocarcinoma lung tissues. Each bar indicates mean ± standard error of mean (SEM; *n* = 5). N, normal lung tissues; GI, grade I adenocarcinoma tissues; GII, grade II adenocarcinoma tissues; GIII, grade III adenocarcinoma tissues (*P < 0.05, compared to normal lung tissues). (**B**) Quantitative PCR (qPCR) analysis of ERGIC3. ERGIC3 downregulated stable cell line (shERGIC3) showed significant differences compared to control (^***^P < 0.001) or shScr (^###^*P* < 0.001) (*n* = 4). (**C**) Real-time cell proliferation analysis using xCELLigence RTCA DP system; 1 × 10^3^ cells from control A549, shScr and shERGIC3 stable cell lines were seeded in 16-well E-plates, and incubated for 72 h (*P < 0.05, compared to control and ^##^*P* < 0.01, compared to shScr, *n* = 3). (**D**) Western blot and densitometric analyses of PCNA in A549 control, shScr and shERGIC3 stable cells (*n* = 4). (**E**) Western blot and densitometric analyses of p-Akt1 (Thr308 and Ser473). Significant differences are indicated by *P < 0.05, **P < 0.01 and ***P < 0.001 (*: compared to control, #: compared to shScr, *n* = 3).

ERGIC3 was over-expressed in human lung adenocarcinoma (Grade I, II and III) compared to normal lung tissues. Twenty human tissue samples (five samples per group) were analyzed using Western blot and densitometry (Figure 1A and Supplementary Figure S1).

### Effect of ERGIC3 knockdown on cell proliferation and Akt1 phosphorylation

**Figure 2:**
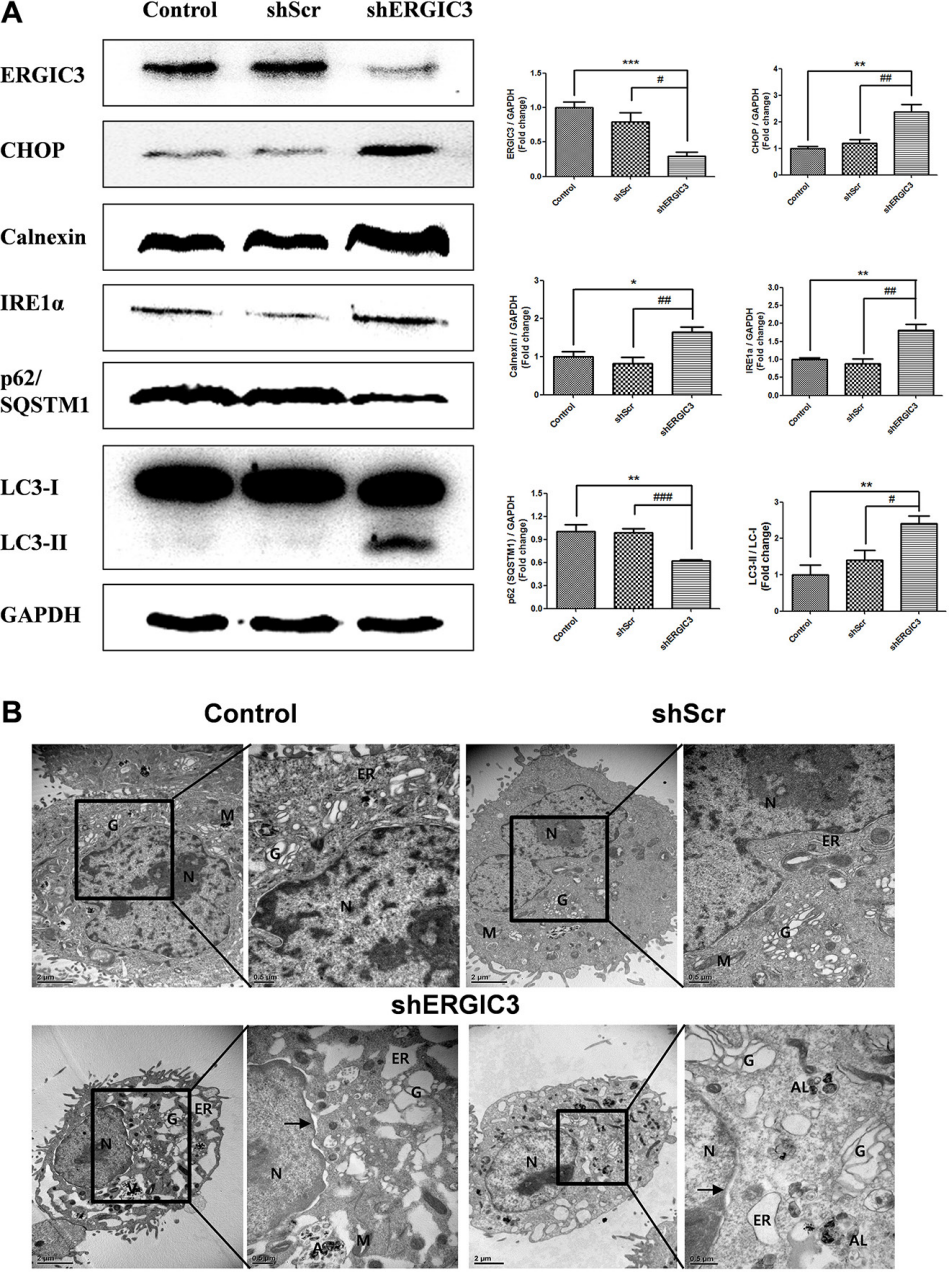
Suppression of ERGIC3 induces ER stress-induced autophagy in A549 cells (**A**) Western blot analyses of ERGIC3, CHOP, Calnexin, IRE1α, p62/SQSTM1 and LC3 in A549 control, shScr and shERGIC3 groups. Cells were cultured for 2 days and then collected for Western blot analysis. Significant differences are indicated by *P < 0.05, **P < 0.01 and ***P < 0.001 (*: compared to control, #: compared to shScr, *n* = 4). (**B**) Subcellular morphological changes in shERGIC3 stable cells were revealed by transmission electron microscopy (TEM) analysis. Cells were cultured for 2 days then were fixed and observed using TEM. In each group of figures, calibration bars in the left figure of each group = 2 μm, while those in the right figure of each group = 0.5 μm. Arrow indicates distorted nucleus. *N*, nucleus; G, Golgi apparatus; ER, endoplasmic reticulum; M, mitochondria; A, autophagosome; AL, Autolysosomes; shScr, small hairpin scramble; shERGIC3, small hairpin ERGIC3.

Over-expression of ERGIC3 in lung cancer tissue led us to study the effect of ERGIC3 knockdown for lung cancer therapy. Sequence number 58 small hairpin RNA (shRNA) targeting ERGIC3 (shERGIC3-58) showed the best silencing efficacy compared to other target sequences (Supplementary Figure S2). Therefore, this target sequence was chosen for further experiments. Stable cell lines expressing scrambled vector (shScr) or shERGIC3 were generated in A549 cells. Significant silencing of ERGIC3 downregulated stable cell line was confirmed by quantitative PCR (qPCR) (Figure 1B) and Western blot (Figure 2A). Using the real-time xCELLigence proliferation detection system, ERGIC3 downregulated stable cells showed a remarkable suppression of cell proliferation compared to control (*P < 0.05) and shScr (^##^*P* < 0.01) groups (Figure 1C). Moreover, proliferating cell nuclear antigen (PCNA) level was also decreased in the shERGIC3 stable cell line (Figure 1D). In addition, Akt1 (protein kinase B) phosphorylation at Thr308 and Ser473 residues was significantly decreased in ERGIC3 downregulated cells (Figure 1E). Therefore, we confirmed that suppression of ERGIC3 decreased Akt1 activation and lung cancer cell proliferation.

### Knockdown of ERGIC3 triggers ER stress-induced autophagy in lung cancer cell lines

Expression of ER stress-related proteins was assessed using Western blot. In the shERGIC3 stable cell line, expression of C/EBP homology protein (CHOP), Calnexin, and inositol-requiring ER to nucleus signal kinase-1α (IRE1α) was up-regulated compared to other control groups. Moreover, induction of autophagy by ERGIC3 downregulation was observed with the increase of the light chain (LC) 3-II to LC3-I ratio. Degradation of p62/ sequestosome-1 (SQSTM1) was induced by an increase in autophagy (Figure 2A). These results were also confirmed by both using another shRNA targeting ERGIC3 (shERGIC3-59, GTGGAACACAACCTGTTCAAGCAACGACT) and another human lung cancer cell line H460 (Supplementary Figure S3). Moreover, after treatment of chloroquine, p62/SQSTM1 was more degraded in shERGIC3 stable cell line compared to shScr (Supplementary Figure S5A).

Dilated ER, swelling of the Golgi apparatus, vacuoles in mitochondria and distorted nuclei were observed in shERGIC3 stable cells in transmission electron microscopy (TEM) images. Engulfed organelles in autophagosomes and autolysosomes were also found in ERGIC3 downregulated stable cell line (Figure 2B, Supplementary Figure S4).

### ERGIC3 is associated with ER and ER stress

**Figure 3:**
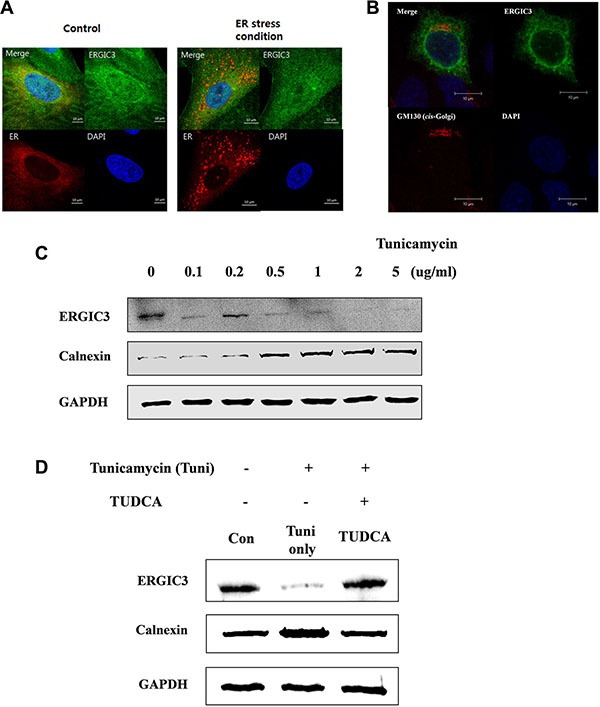
Subcellular localization of ERGIC3 and relation to ER stress (**A**) Confocal laser scanning microscope (CLSM) data analysis of ERGIC3 (Green) and an ER marker (Red). After transfection of the ER marker, tunicamycin (1 μg/mL) was applied for 24 h to A549 cells. After fixation, incubation with an anti-ERGIC3 antibody was performed for 24 h. Calibration bars = 10 μm. Green, ERGIC3; Red, ER marker; Blue, nucleus. (**B**) ERGIC3-tGFP was transfected into A549 cells for 24 h, followed by incubation with anti-GM130 antibody for 24 h, and nuclei staining (DAPI). Calibration bars = 10 μm. Green, ERGIC3; Red, GM130; Blue: nucleus (**C**) Western blot analysis of ERGIC3 and Calnexin expression depending on tunicamycin concentration (0.1 to 5 μg/mL) for 24 h. (**D**) Pre-treatment with 5 mg/mL tauroursodeoxycholic acid (TUDCA) was performed for 2 h, followed by 5 μg/mL tunicamycin for 24 h. Western blot assays of ERGIC3, Calnexin and GAPDH were performed after sampling cell lysates. Con, control; Tuni only, tunicamycin treated; TUDCA, TUDCA pretreated and tunicamycin treated).

To evaluate the potential effect of ERGIC3 on ER and ER stress, we first assessed the localization of ERGIC3 and ER markers in cells in normal and ER stress conditions. As shown in Figure 3, ERGIC3 was localized to the ER in normal condition (control) and partially localized to the ER in ER stress conditions (Figure 3A). Furthermore, ERGIC3 was partially co-localized to the *cis*-Golgi matrix protein 130 (GM130) (Figure 3B). To examine the expression of ERGIC3 in ER stress conditions, A549 cells were treated with various concentrations (0.1–5 μg/mL) of tunicamycin. Expression of ERGIC3 was decreased in ER stress condition induced by tunicamycin (Figure 3C). However, co-treatment with tunicamycin and tauroursodeoxycholic acid (TUDCA; an ER stress inhibitor) did not decrease ERGIC3 expression (Figure 3D). Moreover, treatment of TUDCA inhibits changes of ER stress and autophagy-related proteins in ERGIC3 downregulated cell line compared to TUDCA non-treated cell line (Supplementary Figure S5B).

### ERGIC3 overexpression alleviates ER stress and promotes cell proliferation

Overexpression of ERGIC3 alleviated ER stress induced by tunicamycin treatment. After treatment with tunicamycin, expression of ER stress proteins including IRE1α, CHOP and Calnexin in ERGIC3 overexpressed cells (Tuni Over ER3) was lower compared to the tunicamycin only treated group (Tuni). Moreover, overexpression of ERGIC3 increased PCNA levels (Supplementary Figure S6).

### Aerosol delivery of shERGIC3 suppresses lung cancer by affecting ER stress-induced autophagy, proliferation and angiogenesis in *K-ras^LA1^* mice

To test the silencing efficacy of four targets of mouse ERGIC3 shRNA, Western blot was performed after transfection of shRNAs into LA-4 (mouse lung adenoma) cells. Target sequence A showed the best silencing effect compared to other targets (Supplementary Figure S7); therefore, it was chosen for gene delivery to mice.

**Figure 4:**
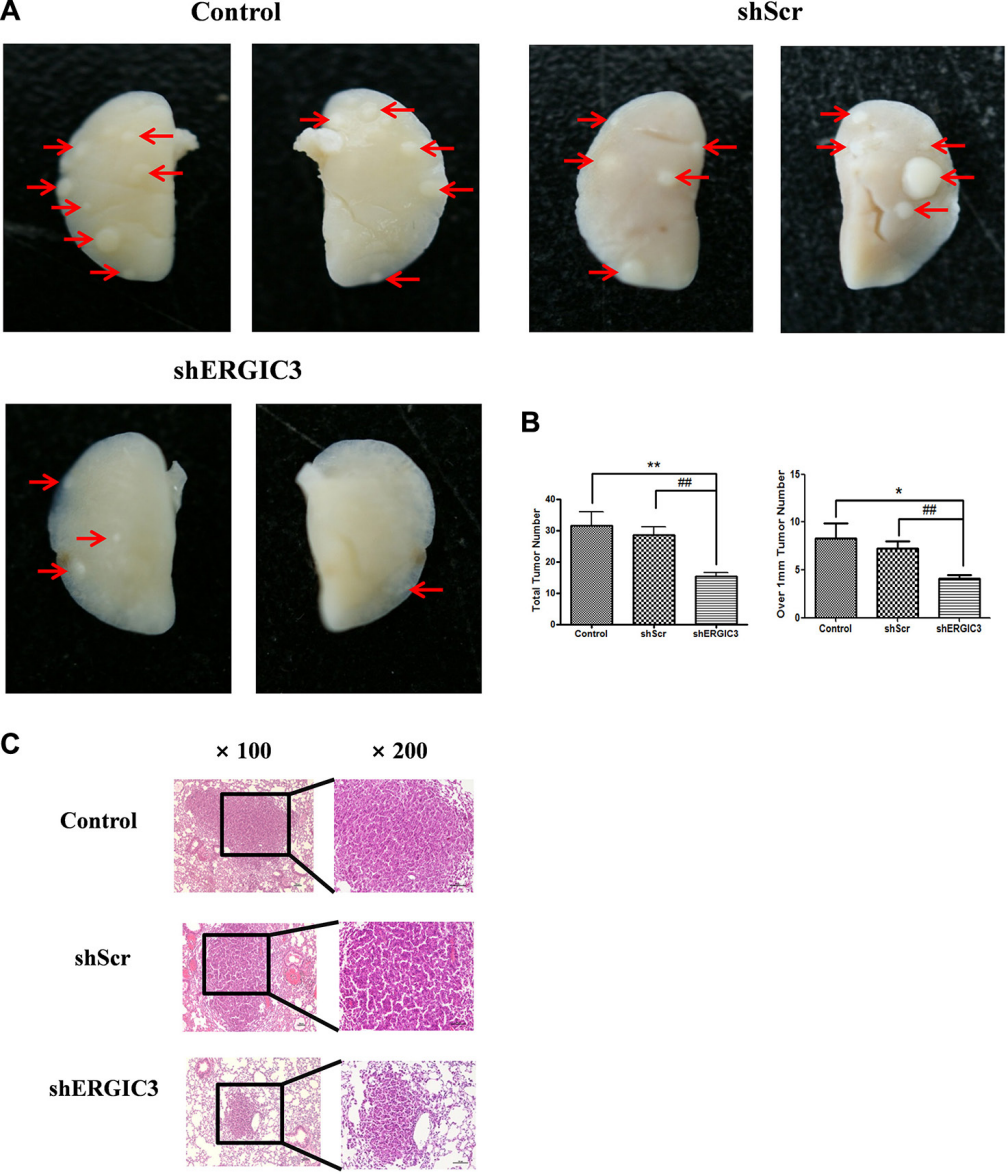
shERGIC3 suppresses lung tumorigenesis in *K-ras^LA1^* mice Aerosols of GPT-SPE/shERGIC3 complexes were delivered to *K-ras^LA1^* mice twice a week for 4 weeks (eight times). (**A**) Figures of lung tumors in control, shScr and shERGIC3-treated *K-ras^LA1^* mice. Arrows indicate tumor lesions. (**B**) Total number of tumors, and tumors over 1 mm in control, shScr and shERGIC3-treated *K-ras^LA1^* mice. Each bar indicates mean ± SEM. Significant differences are indicated by *P < 0.05 and **P < 0.01. (*: compared to control, #: compared to shScr, *n* = 6) (**C**) Histopathological examination of hematoxylin-eosin (H&E) stained lungs of *K-ras^LA1^* mice. Calibration bars = 100 μm.

**Figure 5:**
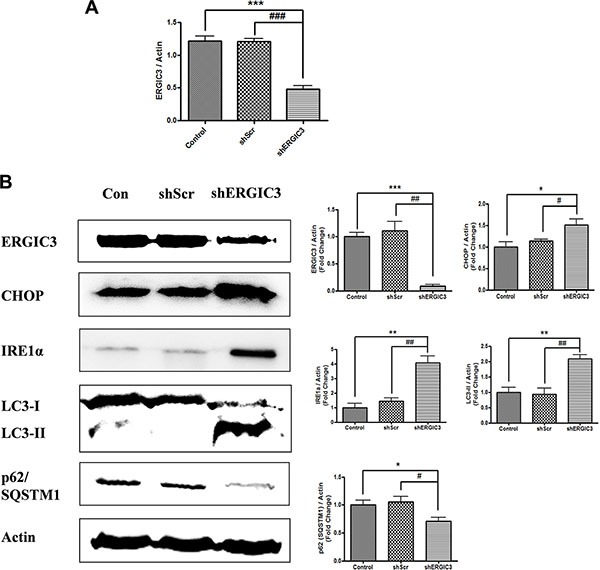
Aerosol delivery of shERGIC3 triggers ER stress-induced autophagy in *K-ras^LA1^* mice (**A**) Quantitative PCR (qPCR) analysis of ERGIC3 in lung tissue. Aerosol delivery of shERGIC3 significantly decreased ERGIC3 expression in the lungs compared to control (***P < 0.001) and shScr (^###^*P* < 0.001) (*n* = 4). (**B**) Western blot and densitometric analyses of ERGIC3, CHOP, IRE1α, LC3 and p62/SQSTM1 in the lungs of control, shScr and shERGIC3-treated mice. Significant differences are indicated by *P < 0.05, **P < 0.01 and ***P < 0.001 (*: compared to control, #: compared to shScr, *n* = 4).

**Figure 6:**
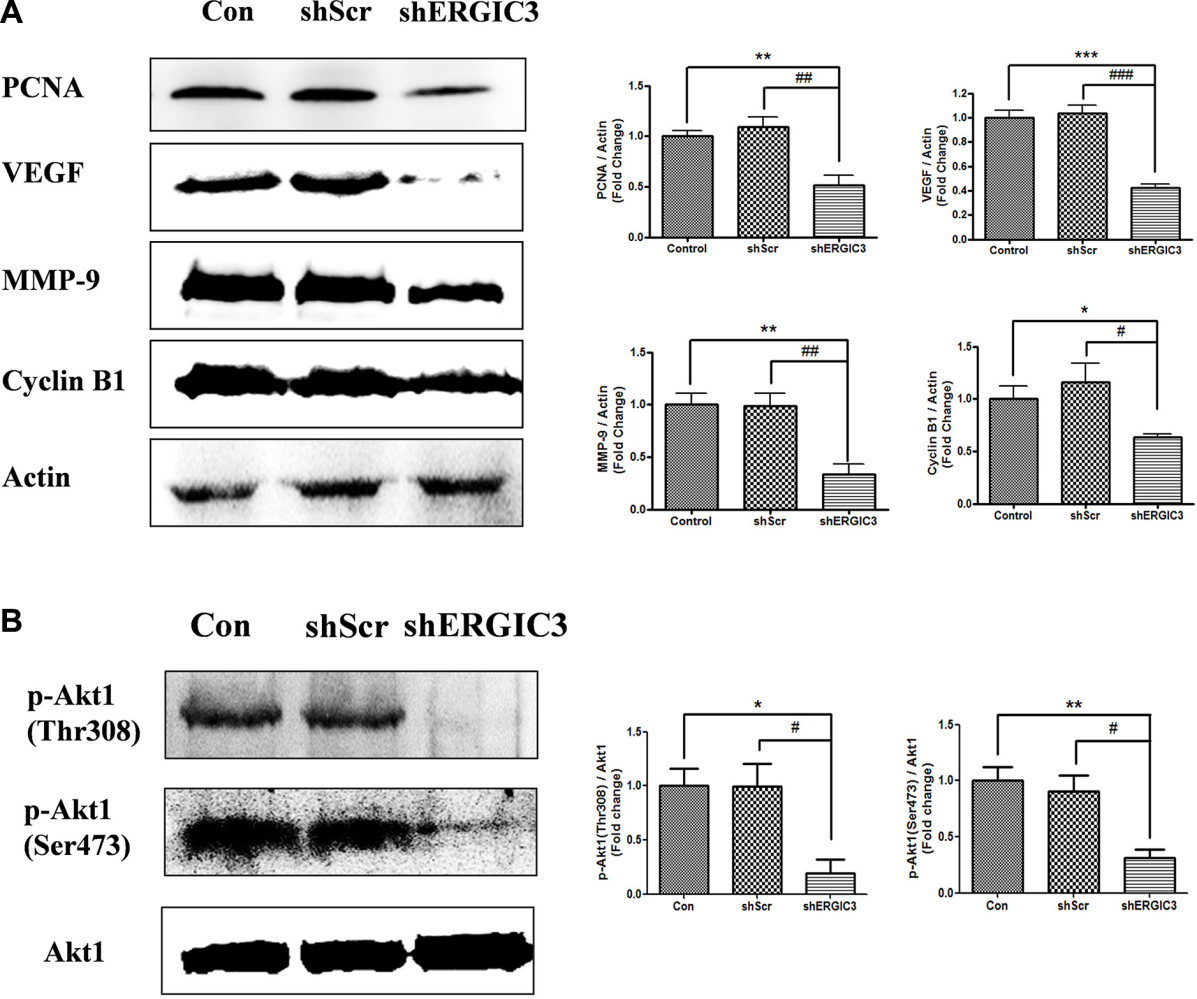
Aerosol delivery of shERGIC3 suppresses proliferation, angiogenesis and Akt1 activation in K-rasLA1 mice (**A**) Western blot and densitometry analyses of PCNA, VEGF, MMP-9 and Cyclin B1 in lungs of control, shScr and shERGIC3-treated mice (*n* = 4). (**B**) Western blot and densitometry analyses of p-Akt1 (Thr308 and Ser473). Statistical significance is indicated by *P < 0.05, **P < 0.01 and ***P < 0.001 (*: compared to control, #: compared to shScr, *n* = 3).

Repeated aerosol delivery of shERGIC3 using glycerol propoxylate triacrylate spermine (GPT-SPE) complexes significantly reduced the total number of tumors as well as tumors larger than 1 mm in *K-ras^LA1^* mice compared to control and shScr-treated groups (Figure 4A, 4B). Significant suppression of ERGIC3 expression in the lungs of mice treated with shERGIC3 was confirmed using qPCR and Western blot (Figure 5). Histological assessment revealed a clear reduction in tumor size, and no adenocarcinoma was observed in the shERGIC3-treated group. Reduced number of adenoma and hyperplasia foci was also observed in the shERGIC3-treated group compared to other control groups (Figure 4C, Table 1). In TEM images of the lungs of shERGIC3-treated mice, dilated ER and ingested organelles in autophagosomes were observed (Supplementary Figure S8). To assess whether aerosol delivery of shERGIC3 stimulated ER stress-induced autophagy in *K-ras^LA1^* mice, ER stress- and autophagy-related proteins were analyzed. CHOP and IRE1α expression levels increased as a result of ERGIC3 suppression. LC3-II expression also increased, while p62 level decreased because of autophagy in the lung tissues of mice treated with shERGIC3 (Figure 5B). The expression of matrix metalloproteinase-9 (MMP-9), proliferating cell nuclear antigen (PCNA), vascular endothelial growth factor (VEGF) and cyclin B1 was also significantly decreased in the shERGIC3-treated group compared to other control groups (Figure 6A). Decreased Akt1 phosphorylation (residues Thr308 and Ser473) was also observed in the lungs of shERGIC3-treated mice, similarly to the *in vitro* studies (Figure 6B).

**Table 1:** Number of tumor incidences in the left lungs of *K-ras* mice

Group	Mice identification	No. of Adenocarcinoma	No. of Adenoma	No. of hyperplasia foci
Con	1	0	1	2
	2	0	2	4
	3	1	0	9
	4	0	1	4
	5	1	0	3
	6	1	0	3
Avg		0.5	0.667	4.167
STD		0.548	0.816	2.483
shScr	1	1	1	3
	2	1	1	3
	3	1	1	4
	4	0	1	6
	5	0	2	6
	6	2	0	5
Avg		0.833	1	4.5
STD		0.753	0.632	1.378
shERGIC3	1	0	0	2
	2	0	1	0
	3	0	0	2
	4	0	0	4
	5	0	1	1
	6	0	0	3
Avg		0	0.333	2
STD		0	0.516	1.414

Con: control group (not treated); shScr: shScramble control-treated group; shERGIC3: shERGIC3-treated group.

## DISCUSSION

Cancer statistics show that lung cancer causes the highest number of estimated deaths in the United States [15]. Gene therapy is a promising and efficient approach for the suppression of tumorigenesis by directly influencing the defective genes [16]. Moreover, this therapy has been applied to various diseases, and the efficiency of delivery is essential for the success of this therapy [17, 18]. Aerosol delivery enables non-invasive and efficient delivery of macromolecules to the lung [19, 20]. Moreover, aerosol delivery using various cationic carriers and viral vectors has been successfully used in lung cancer therapy [21]. We previously developed a GPT-SPE cationic polymer which showed biocompatibility and high transfection efficiency [22]. Therefore in this study, GPT-SPE cationic carrier was used for aerosol delivery of shERGIC3 to *K-ras^LA1^* mice.

In cancer cell, ER to Golgi trafficking is elevated, therefore, the ER to Golgi network has emerged as a potential target for cancer treatment [2, 23]. Our data shows that ERGIC3 is localized to ER and *cis*-Golgi (Figure 3A, 3B). Some binding partner proteins of ERGIC3 are also localized to ER and Golgi apparatus (Supplementary Table S1) Moreover, knockdown of ERGIC3 suppressed proliferation and tumor growth (Figures 1, 4). Although the role of ERGIC3 in membrane trafficking still needs to be proven, our results may indicate the relationship between ERGIC3 and ER to Golgi transport.

In our study, we focused on the anti-cancer effect of the ERGIC3 downregulation. Suppression of ERGIC3 increased ER stress-related proteins and LC3-II levels (Figure 2A). Furthermore, treatment of tunicamycin induced suppression of ERGIC3 and increase of Calnexin expressions. Increase of ER stress proteins may be due to the decrease of the ERGIC3 expression under ER stress condition (Figure 3C). Moreover, ER dilation, Golgi swelling, autophagosomes and autolysosomes were found in the ERGIC3 downregulated stable cell line (Figure 2B and Supplementary Figure S3). These results show that downregulation of ERGIC3 induces autophagy and ER stress in lung cancer cells.

Autophagy is a major intracellular process that degrades defective cytoplasmic organelles or protein aggregates via lysosomal machinery, and is also associated with various pathological processes [24, 25]. Autophagy not only facilitates cell survival, it can also suppress tumor development. This dual function of autophagy is still controversial; however, if cellular stress is too high, an increase in autophagic flux can induce cell death [26]. Autophagic cell death is also involved in cancer cell death. Whereas reduced autophagy function is related to tumorigenesis [27] Autophagy can act as a tumor suppressor by limiting cell growth or inducing cell death [28]. Although the complex roles of autophagy need to be elucidated, we can consider activation of autophagy as a therapeutic target in cancer treatment. The p62 protein (also known as SQSTM1), is an autophagy substrate, and an increase in its expression has been demonstrated in various cancers [26, 29]. The p62/SQSTM1 protein accumulates because of defective autophagy, and this is directly linked to tumorigenesis. Therefore, control of p62/SQSTM1 is crucial for cancer therapy [30, 31]. The absence of p62/SQSTM1 inhibits *ras*-induced lung adenocarcinomas and increased cell death [32]. In this study, suppression of ERGIC3 increased LC3-II levels, and reduced p62 expression both *in vitro* and *in vivo* (Figures 2A, 5B).

UPR is activated under ER stress, and is the subcellular response for the reestablishment of ER function and adaptation to a changed environment [33]. In human lung cancer lesions, expression of ER stress proteins is linked to autophagy-related proteins such as LC3 and Beclin-1 [34]. ER stress is also an essential inducer of pre-autophagosomal structures, LC3 conversion and autophagy [35, 36]. Moreover, ER stress-induced autophagy is activated through IRE1α mediation [14]. In our data, stimulation of ER stress in ERGIC3 knockdown status was confirmed by an increase in related proteins (i.e., IRE1α and CHOP) *in vitro* and *in vivo*. Moreover, shERGIC3 triggered autophagy and autophagosome formation (Figures 2, 5B). Therefore, we can conclude that knockdown of ERGIC3 stimulates ER stress-induced autophagy.

Prolonged and severe ER stress can induce cell death [33]. Additionally, ER stress can induce autophagic cell death [37, 38]. ER stress-inducing reagents including tunicamycin and dithiothreitol (DTT) also aggravate autophagy and cell death [39]. Our data showed that aerosol delivery of shERGIC3 inhibited lung tumorigenesis in *K-ras^LA1^* mice through ER stress-induced autophagy (Figures 4, 5). The number of tumors was also decreased and no adenocarcinoma was observed in shERGIC3-treated mice (Table 1).

Activation of Akt1 is associated with the proliferation, angiogenesis, growth and survival of cancer cells. Akt1 activation is induced by phosphorylation at Thr308 and Ser473 residues [40]. Whereas inhibition of Akt1 can promote the induction of fatal ER stress and autophagy mediated cell death [41–44]. Moreover, ER stress-induced autophagy is attributed to the suppression of AKT/TSC/mTOR pathway [39]. Our data show that suppression of ERGIC3 inhibited Akt1 phosphorylation at Thr308 and Ser473 residues (Figures 1E, 6B). Increased ER stress-induced autophagy was also observed in ERGIC3 downregulated stable cell line (Figures 2, 5B). Therefore, depletion of ERGIC3 suppresses Akt1 activation as downstream of the ER stress induction. In addition, according to our analysis of proteomics, downregulation of ERGIC3 induced suppression of the cell cycle, cytokinesis and cell proliferation (Supplementary Figure S9).

In conclusion, our data showed that knockdown of ERGIC3 triggers ER stress-induced autophagic cancer cell death and the suppression of Akt1 activation (Supplementary Figure S10). Moreover, suppression of ERGIC3 is associated with the expression of cell cycle- and mitosis-related proteins. In addition to lung cancer, ERGIC3 is also associated with hepatocellular carcinomas, and its expression is enhanced in colorectal tumors [4, 6]. Therefore, ERGIC3 could be a novel target and marker for various cancers and cancer gene therapy.

## MATERIALS AND METHODS

### Materials

Antibodies against CHOP, IRE1α, Calnexin, LC3, GM130, p-Akt1(Thr308) and p-Akt1(Ser473) were purchased from Cell Signaling Technology (Boston, MA, USA). ERGIC3, p62 and MMP-9 antibodies were obtained from Abcam (Beverly, MA, USA). Antibodies against glyceraldehyde 3-phosphate dehydrogenase (GAPDH) and Akt1 were purchased from AbFrontier (Seoul, Korea). Antibodies against PCNA, VEGF, cyclin B1 and actin were purchased from Santa Cruz Biotechnology (Santa Cruz, CA, USA). Tunicamycin and chloroquine were purchased from Sigma (St. Louis, MO, USA). TUDCA was purchased from EMD Chemicals (Gibbstown, NJ, USA).

### Plasmids and shRNA target sequences

Plasmids were propagated in *E. coli*, extracted and purified using a Labopass plasmid DNA purification kit (Cosmo Genetech, Seoul, Korea). Full-length human ERGIC3 (GenBank ID:NM_015966.2) was subcloned in pCMV6-AC-GFP (OriGene Technologies Inc., Rockville, MD, USA), pCMV-Myc (Clontech Laboratories Inc., Palo Alto, CA, USA) or pGBKT7 (Clontech) plasmid vectors. ERGIC3 shRNAs were purchased from OriGene.

### Cell culture and generation of ERGIC3 downregulated stable cell line

A549, H460 and LA-4 cell lines were obtained from American Type Culture Collection (Rockville, MD, USA). A549 and H460 cells were cultured in F-12 medium (GIBCO BRL Life Technologies Inc., Gaithersburg, MD, USA) and RPMI medium (HyClone, Logan, UT, USA), respectively, with 10% fetal bovine serum (FBS) and 1% penicillin/streptomycin (GibcoBRL, Grand Island, NY, USA). LA-4 cells were cultured in RPMI medium (HyClone) with 15% FBS and 1% penicillin/streptomycin. For the generation of a downregulated ERGIC3 stable cell line, 1 × 10^6^ cells were cultured in a T75 flask. After cell stabilization, plasmids were transfected using TransIT^R^-LT1 (Mirus Bio Corp., Madison, WI, USA) and cells were selected using 1 μl/mL puromycin (InvivoGen, San Diego, CA, USA).

### Western blot analysis

Human lung tissue samples were obtained from the Korea Lung Tissue Bank (KLTB, Seoul, Korea). All experiments involving human tissues were authorized by the Seoul National University Institutional Review Board (SNUIRB-E1201/001-001) and KLTB (KU Guro Gene Bank 2012-004). Lung lobes were homogenized using 300 μL of a 2.5× Passive Lysis Buffer (Promega, Madison, WI, USA), while A549 cells were lysed using 1× Cell Culture Lysis Buffer (Promega). Immunoblotting was performed by incubating with the primary antibody (1:2500) at 4°C overnight, and then with a horseradish peroxidase (HRP)-conjugated secondary antibody (1:2000; Invitrogen, Carlsbad, CA, USA) for 3 h at room temperature (RT). Bands were detected using a luminescent image detector (Ez-Capture MG), and analysis was performed using the CS Analyzer program (ATTO, Tokyo, Japan).

### *In vivo* Aerosol delivery of GPT-SPE/shERGIC3 complex

Animals were maintained under the animal guidelines of the Seoul National University, and all animal experiments were reviewed and approved by the Institutional Animal Care and Use Committee of Seoul National University (SNU-130710-4). *K-ras^LA1^* (non-small cell murine lung cancer model) mice were obtained from the Human Cancer Consortium-National Cancer Institute (Frederick, MD, USA). Ten-week-old male *K-ras^LA1^* mice were divided into the following three groups (6 mice per group, total 18 mice); control, small hairpin scramble vector control and shERGIC3-treated groups. Mice in the control (Con) group were untreated, while mice in the other two groups were treated with aerosol containing 8 mg GPT-SPE and 0.8 mg shRNA (small hairpin ERGIC3 or small hairpin scramble) twice a week for 4 weeks. GPT-SPE is a spermine-based biocompatible DNA carrier which can be used in shRNA-based lung cancer therapy [22]. Mice were treated with aerosol containing GPT-SPE/shERGIC3 or GPT-SPE/shScr complex in a nose-only exposure chamber. Aerosol was generated by a nebulizer (Korean patent #20304964). Tumor lesions on the entire lung surface were carefully counted, and the diameter of the tumors was also measured using a digital caliper. Left lung lobes were collected for histopathological analysis. After necropsy, remaining lung lobes were stored at −70°C for Western blot and RNA analysis.

### Histopathology of lung tissues

Left lung lobes were fixed in 10% neutral-buffered formalin, and embedded in paraffin blocks. Blocks were sectioned at 5 μm thickness, and sections were stained with hematoxylin and eosin (H&E, Sigma) for histological analysis.

### Transmission electron microscope (TEM)

Mice lung tissues, A549 and stable cell lines were fixed in 2.5% glutaraldehyde (EMS, Hatfield, PA, USA) overnight. Samples were carefully washed and stained using 1% osmium tetroxide solution (EMS) for 2 h at 4°C. Samples were dehydrated with a gradient of ethanol and infiltrated in a 1:1 propylene oxide Epon resin (EMS) mixture. Then, samples were embedded in Epon resin and polymerized for 24 h at 70°C. Ultrathin sections (40–70 nm thick) were obtained on an ultramicrotome (Leica, Nussloch, Germany), and mounted on copper grids. Finally, sectioned samples were counterstained with lead citrate and uranyl acetate. Samples were observed using a transmission electron microscope (JEOL, Tokyo, Japan).

### Plasmid transfection, immunostaining and DAPI stain

For plasmid transfection, 1 × 10^4^ of A549 cells were cultured in two-well chamber slides. ERGIC3-TurboGFP or ERGIC3-Myc plasmid vectors were transfected into these cells using Metafectene Pro (Biontex Laboratories, Martinsried, Germany) according to the manufacturer's protocol. CellLight^™^ ER-RFP BacMan 2.0 (an ER marker) was purchased from Invitrogen (Carlsbad, CA, USA).

For immunostaining, slides were washed in phosphate buffered-saline (PBS), fixed in 3% paraformaldehyde (PFA) solution for 5 min, and post fixed in a 4% PFA solution for 10 min. Samples were incubated in 3% BSA and 0.1% saponin (Sigma) in PBS for 1h at RT, for the blocking of non-specific binding sites. Primary antibodies (diluted 1:250) were incubated overnight at 4°C. After washing, cells were incubated with secondary antibodies (diluted 1:500) conjugated to Alexa Fluor 488 or 555 (Invitrogen) for 1h at RT. Slides were washed in PBS and cover slipped using Fluoroshield^™^ (Sigma) for DAPI staining. Slides were observed using a confocal laser scanning microscope (LSM710, Carl Zeiss GmbH, Jena, Germany).

### Cell proliferation analysis

Real-time cell proliferation analysis was performed using xCELLigence RTCA DP system (Roche Applied Science, Indianapolis, IN, USA). This device analyzes real-time cell proliferation through measurement of the electrical impedance of microelectrodes integrated in the bottom of plates. For analysis, 1.5 × 10^3^ A549 cells were seeded in 16-well E-plates and incubated for 72 h.

### Quantitative real-time PCR

Total RNA was isolated from A549 cells and accessory lung lobes of *K-ras^LA1^* mice using the QuickGene RNA kit (Fujifilm's Life Science System, Tokyo, Japan). Reverse transcription was performed using SuPrimedScript RT Premix (GeNet Bio, Cheonan, Korea) for conversion to cDNA. Reverse transcription was carried out at 25°C for 10 min, 37°C for 30 min, and 85°C for 5 min. Quantitative real-time PCR was performed using the CFX96^™^ Real-Time System (Bio-Rad, Richmond, CA, USA), and cDNA was amplified using Prime Q-mastermix (GeNet Bio) and specific primers following the method and conditions used for previously described quantitative real-time PCR experiments [45]. The result was analyzed using Bio-Rad CFX Manager Version 2.1 software (Bio-Rad). Sequences of primers were as follows: human ERGIC3, forward (5′-TCGCTGTGAGAGCTGCTATG-3′) and reverse (5′-C GCACATCTTCACAGGTGTT-3′); human GAPDH, forward (5′-GCCCAATACGACCAAATC -3′) and reverse (5′-ACTCAGCCGCATCTT-3′); mouse ERGIC3, forward (5′-CCACAGTGTACATGAAGGTGGA-3′) and reverse (5′-GAGCTCATACAGCACAAAGACC-3′); mouse Actin, forward (5′-TTTCCAGCCTTCCTTCTTGGGTATG-3′) and reverse (5′-CACTGTGTTGGCATAGAGGTCTTTAC-3′).

### Statistical analyses

Statistical significances of difference were analyzed using Microcal Origin student's *t*-test two populations (Microcal Software, Northampton, MA, USA). The significances were set by probability values (*P < 0.05, ** *P* < 0.01 and ***P < 0.001) compared to the corresponding values. Each bar in the graphs indicates the mean ± standard error of mean (SEM).

## SUPPLEMENTARY MATERIALS FIGURES AND TABLE



## References

[1] Appenzeller-Herzog C, Hauri HP (2006). The ER-Golgi intermediate compartment (ERGIC): in search of its identity and function. J Cell Sci.

[2] Wlodkowic D, Skommer J, McGuinness D, Hillier C, Darzynkiewicz Z (2009). ER-Golgi network--a future target for anti-cancer therapy. Leuk Res.

[3] Nishikawa M, Kira Y, Yabunaka Y, Inoue M (2007). Identification and characterization of endoplasmic reticulum-associated protein, ERp43. Gene.

[4] Zhang LY, Liu M, Li X, Tang H (2013). miR-490-3p modulates cell growth and epithelial to mesenchymal transition of hepatocellular carcinoma cells by targeting endoplasmic reticulum-Golgi intermediate compartment protein 3 (ERGIC3). J Biol Chem.

[5] Wu M, Tu T, Huang Y, Cao Y (2013). Suppression subtractive hybridization identified differentially expressed genes in lung adenocarcinoma: ERGIC3 as a novel lung cancer-related gene. BMC Cancer.

[6] Brim H, Abu-Asab MS, Nouraie M, Salazar J, Deleo J, Razjouyan H, Mokarram P, Schaffer AA, Naghibhossaini F, Ashktorab H (2014). An integrative CGH, MSI and candidate genes methylation analysis of colorectal tumors. PLoS One.

[7] Gething MJ, Sambrook J (1992). Protein folding in the cell. Nature.

[8] Kaufman RJ (1999). Stress signaling from the lumen of the endoplasmic reticulum: coordination of gene transcriptional and translational controls. Genes Dev.

[9] Mori K (2000). Tripartite management of unfolded proteins in the endoplasmic reticulum. Cell.

[10] Zhang J, Morris MW, Dorsett-Martin WA, Drake LC, Anderson CD (2013). Autophagy is involved in endoplasmic reticulum stress-induced cell death of rat hepatocytes. J Surg Res.

[11] Verfaillie T, Salazar M, Velasco G, Agostinis P, Linking ER (2010). Stress to Autophagy: Potential Implications for Cancer Therapy. Int J Cell Biol.

[12] Mizushima N, Levine B, Cuervo AM, Klionsky DJ (2008). Autophagy fights disease through cellular self-digestion. Nature.

[13] Schroder M, Kaufman RJ (2005). The mammalian unfolded protein response. Annu Rev Biochem.

[14] Ding WX, Ni HM, Gao W, Yoshimori T, Stolz DB, Ron D, Yin XM (2007). Linking of autophagy to ubiquitin-proteasome system is important for the regulation of endoplasmic reticulum stress and cell viability. Am J Pathol.

[15] Siegel R, Ma J, Zou Z, Jemal A (2014). Cancer statistics 2014. CA Cancer J Clin.

[16] Merdan T, Kopecek J, Kissel T (2002). Prospects for cationic polymers in gene and oligonucleotide therapy against cancer. Adv Drug Deliv Rev.

[17] Koehler DR, Hitt MM, Hu J (2001). Challenges and strategies for cystic fibrosis lung gene therapy. Mol Therapy.

[18] Cai K, Sham M, Tam P, Lam WK, Xu R (2003). Lung cancer gene therapy. Gene Ther Mol Biol.

[19] Stribling R, Brunette E, Liggitt D, Gaensler K, Debs R (1992). Aerosol gene delivery *in vivo*. Proc Natl Acad Sci USA.

[20] Brain JD, Valberg PA (1979). Deposition of aerosol in the respiratory tract. Am Rev Respir Dis.

[21] Hong SH, Park SJ, Lee S, Cho CS, Cho MH (2014). Aerosol gene delivery using viral vectors and cationic carriers for *in vivo* lung cancer therapy. Expert Opin Drug Deliv.

[22] Jiang HL, Hong SH, Kim YK, Islam MA, Kim HJ, Choi YJ, Nah JW, Lee KH, Han KW, Chae C, Cho CS, Cho MH (2011). Aerosol delivery of spermine-based poly(amino ester)/Akt1 shRNA complexes for lung cancer gene therapy. Int J Pharm.

[23] Boelens J, Lust S, Offner F, Bracke ME, Vanhoecke BW (2007). The endoplasmic reticulum: a target for new anticancer drugs. In Vivo.

[24] Mizushima N, Yoshimori T, Levine B (2010). Methods in mammalian autophagy research. Cell.

[25] Mizushima N (2004). Methods for monitoring autophagy. Int J Biochem Cell Biol.

[26] Hoare M, Young AR, Narita M (2011). Autophagy in cancer: having your cake and eating it. Semin Cancer Biol.

[27] Levine B (2007). Cell biology: autophagy and cancer. Nature.

[28] Shintani T, Klionsky DJ (2004). Autophagy in health and disease: a double-edged sword. Science.

[29] Bjørkøy G, Lamark T, Brech A, Outzen H, Perander M, Overvatn A, Stenmark H, Johansen T (2005). p62/SQSTM1 forms protein aggregates degraded by autophagy and has a protective effect on huntingtin-induced cell death. J Cell Biol.

[30] Mathew R, Karp CM, Beaudoin B, Vuong N, Chen G, Chen HY, Bray K, Reddy A, Bhanot G, Gelinas C, Dipaola RS, Karantza-Wadsworth V, White E (2009). Autophagy suppresses tumorigenesis through elimination of p62. Cell.

[31] Moscat J, Diaz-Meco MT (2009). p62 at the crossroads of autophagy, apoptosis, and cancer. Cell.

[32] Duran A, Linares JF, Galvez AS, Wikenheiser K, Flores JM, Diaz-Meco MT, Moscat J (2008). The signaling adaptor p62 is an important NF-kappaB mediator in tumorigenesis. Cancer cell.

[33] Xu C, Bailly-Maitre B, Reed JC (2005). Endoplasmic reticulum stress: cell life and death decisions. J Clin Invest.

[34] Kim KM, Yu TK, Chu HH, Park HS, Jang KY, Moon WS, Kang MJ, Lee DG, Kim MH, Lee JH, Chung MJ (2012). Expression of ER stress and autophagy-related molecules in human non-small cell lung cancer and premalignant lesions. Int J Cancer.

[35] Yorimitsu T, Nair U, Yang Z, Klionsky DJ (2006). Endoplasmic reticulum stress triggers autophagy. J Biol Chem.

[36] Kouroku Y, Fujita E, Tanida I, Ueno T, Isoai A, Kumagai H, Ogawa S, Kaufman RJ, Kominami E, Momoi T (2007). ER stress (PERK/eIF2alpha phosphorylation) mediates the polyglutamine-induced LC3 conversion, an essential step for autophagy formation. Cell Death Differ.

[37] Gozuacik D, Bialik S, Raveh T, Mitou G, Shohat G, Sabanay H, Mizushima N, Yoshimori T, Kimchi A (2008). DAP-kinase is a mediator of endoplasmic reticulum stress-induced caspase activation and autophagic cell death. Cell Death Differ.

[38] Ding WX, Ni HM, Gao W, Hou YF, Melan MA, Chen X, Stolz DB, Shao ZM, Yin XM (2007). Differential effects of endoplasmic reticulum stress-induced autophagy on cell survival. J Biological chemistry.

[39] Qin L, Wang Z, Tao L, Wang Y (2010). ER stress negatively regulates AKT/TSC/mTOR pathway to enhance autophagy. Autophagy.

[40] Vivanco I, Sawyers CL (2002). The phosphatidylinositol 3-Kinase AKT pathway in human cancer. Nat Rev Cancer.

[41] Gurpinar E, Grizzle WE, Shacka JJ, Mader BJ, Li N, Piazza NA, Russo S, Keeton AB, Piazza (2013). A novel sulindac derivative inhibits lung adenocarcinoma cell growth through suppression of Akt/mTOR signaling and induction of autophagy. Mol Cancer Ther.

[42] Mimura N, Hideshima T, Shimomura T, Suzuki R, Ohguchi H, Rizq O, Kikuchi S, Yoshida Y, Cottini F, Jakubikova J, Cirstea D, Gorgun G, Minami J (2014). Selective and potent Akt inhibition triggers anti-myeloma activities and enhances fatal endoplasmic reticulum stress induced by proteasome inhibition. Cancer Res.

[43] Zhai B, Hu F, Jiang X, Xu J, Zhao D, Liu B, Pan S, Dong X, Tan G, Wei Z, Qiao H, Jiang H, Sun X (2014). Inhibition of Akt reverses the acquired resistance to sorafenib by switching protective autophagy to autophagic cell death in hepatocellular carcinoma. Mol Cancer Ther.

[44] Salazar M, Carracedo A, Salanueva IJ, Hernández-Tiedra S, Lorente M, Egia A, Vázquez P, Blázquez C, Torres S, García S, Nowak J, Fimia GM, Piacentini M (2009). Cannabinoid action induces autophagy-mediated cell death through stimulation of ER stress in human glioma cells. J Clin Invest.

[45] Chang SH, Hong SH, Jiang HL, Minai-Tehrani A, Yu KN, Lee JH, Kim JE, Shin JY, Kang B, Park S, Han K, Chae C, Cho MH (2012). GOLGA2/GM130, cis-Golgi matrix protein, is a novel target of anticancer gene therapy. Mol Ther.

