# Supplementary material for: Endoplasmic reticulum-Golgi intermediate compartment protein 3 knockdown suppresses lung cancer through endoplasmic reticulum stress-induced autophagy
Source: Oncotarget. 2016 Aug 29;7(40):65335–47. doi: 10.18632/oncotarget.11678 (PMC5323159; doi:10.18632/oncotarget.11678)
Supplement: Supplementary file 1 [file oncotarget-07-65335-s001.pdf]

Endoplasmic reticulum-Golgi intermediate compartment protein 3 knockdown suppresses lung cancer through endoplasmic reticulum stress-induced autophagy

Supplementary Materials

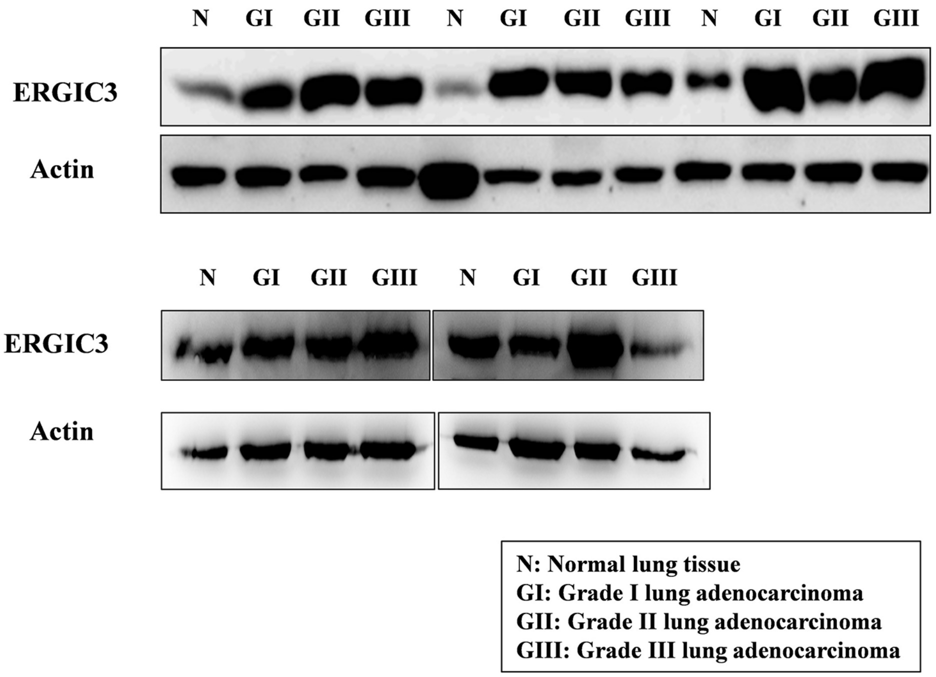

Supplementary Figure S1: ERGIC3 expression in human normal and lung adenocarcinoma tissues. Western blot analysis of ERGIC3. (N, normal lung tissue; GI, grade I adenocarcinoma; GII, grade II adenocarcinoma; GIII, grade III adenocarcinoma).

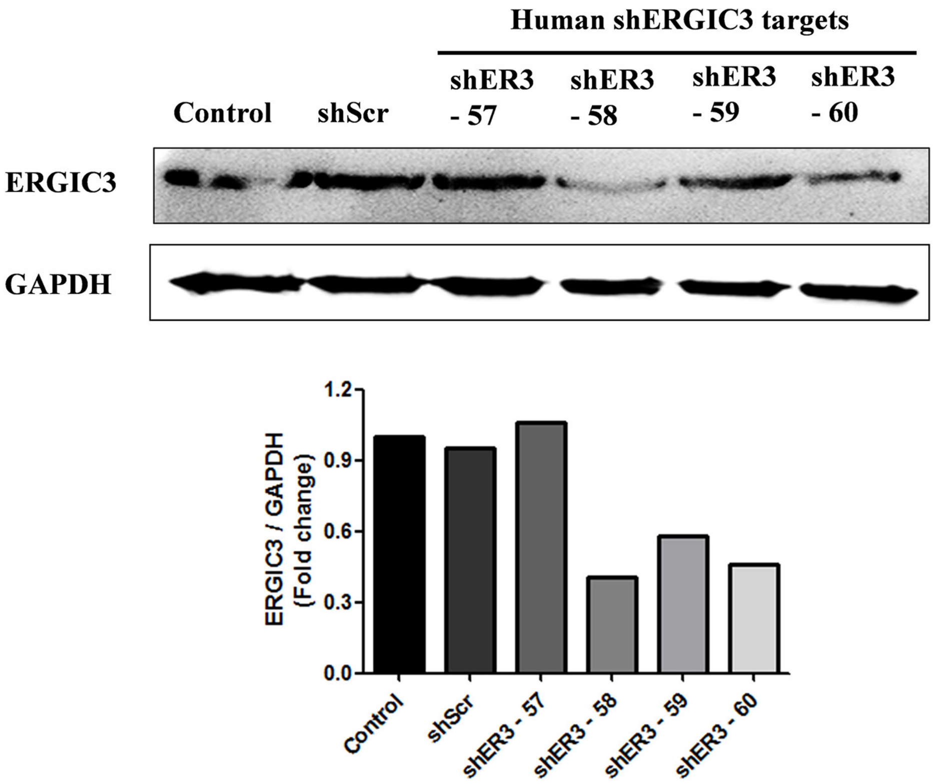

Supplementary Figure S2: Efficiencies of various target sequences and effect of human small hairpin on ERGIC3 expression. Western blot was performed after transfection for 48h in A549 cells. Target number 57, ACCGTGACCATTGTCAGTGGCCTTCTCAT; target number 58, GCAGAAGAATGAAGGCTGCCAGGTGTATG; target number 59, GTGGAACACAACCTGTTCAAGCAACGACT; target number 60, CTACCACTCAGCACGAGCCATCCAGAAGA. Densitometric analysis was performed for the ratio of ERGIC3 to GAPDH. shScr, small hairpin RNA scramble; shER3, small hairpin RNA of ERGIC3.

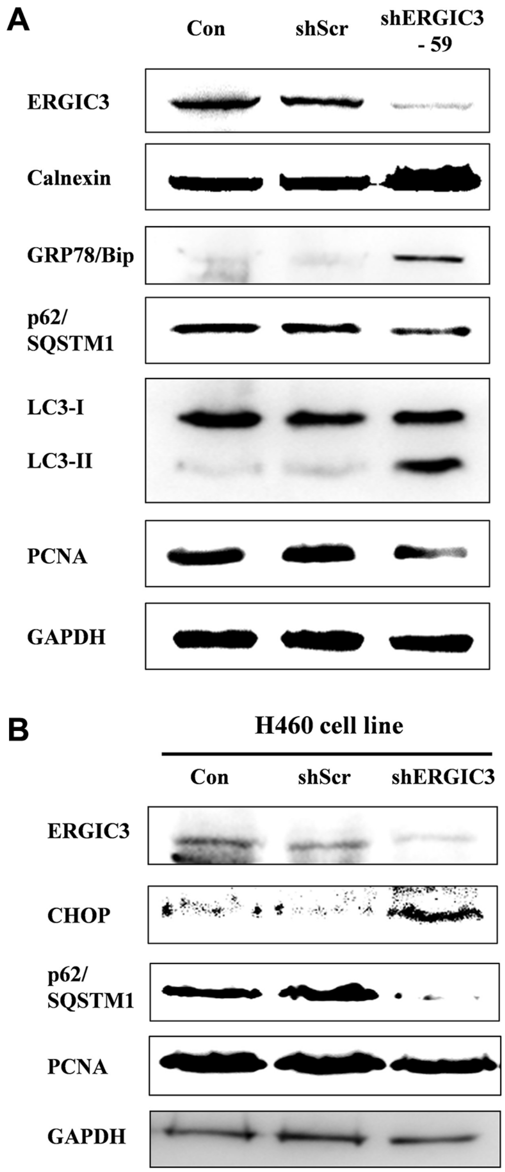

Supplementary Figure S3: Confirmation of role of ERGIC3 in ER stress-induced autophagy in H460 cells or stable cell line using another shRNA targeting ERGIC3 (shERGIC3-59) in A549 cells. (**A**) Western blot of ERGIC3, Calnexin, GRP78/Bip, p62/SQSTM1, LC3 and PCNA in A549 control, shScr and shERGIC3-59 (target number 59: GTGGAACACAACCTGTTCAAGCAACGACT) stable cell line. (**B**) Western blot of ERGIC3, CHOP, p62/SQSTM1 and PCNA in H460 control, shScr and shERGIC3 stable cells. Cells were cultured for 2 days and then collected for Western blot. Con, control; shScr, small hairpin scramble; shERGIC3, small hairpin ERGIC3 stable cell line.

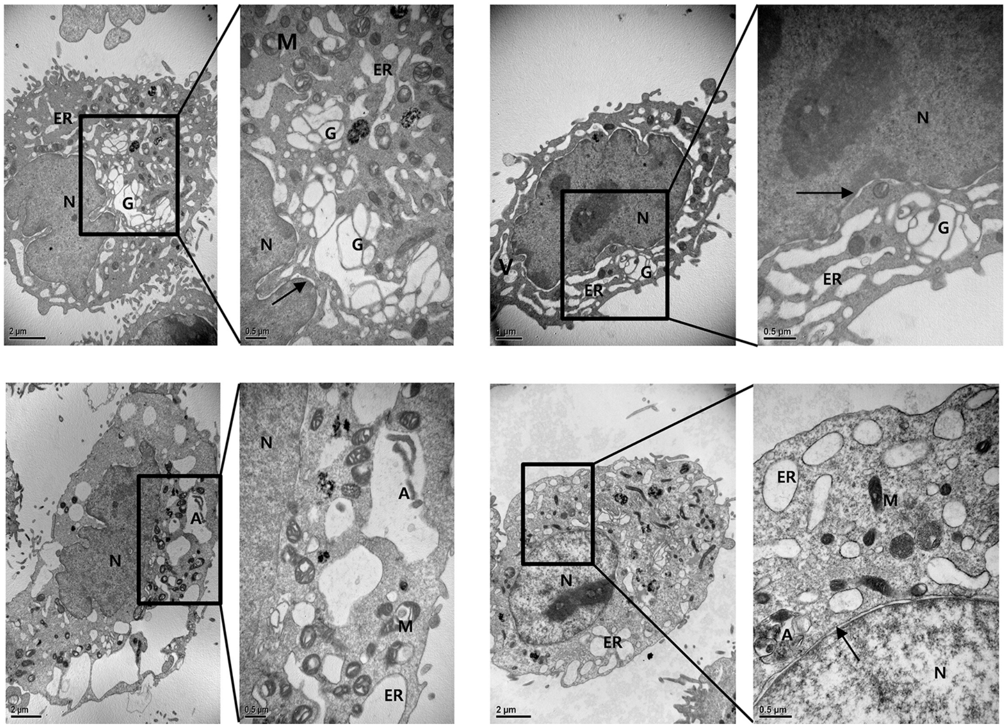

Supplementary Figure S4: ER stress-induced autophagy in shERGIC3 stable cell line. shERGIC3 stable cells were cultured for 2 days, and fixed for transmission electron microscopy. In each paired figures, calibration bar in the left figure = 2 μm, while in the right figure = 0.5 μm. Arrows indicate distorted nucleus. (N, nucleus; G, Golgi apparatus; ER, endoplasmic reticulum; M, mitochondria; A, autophagosome).

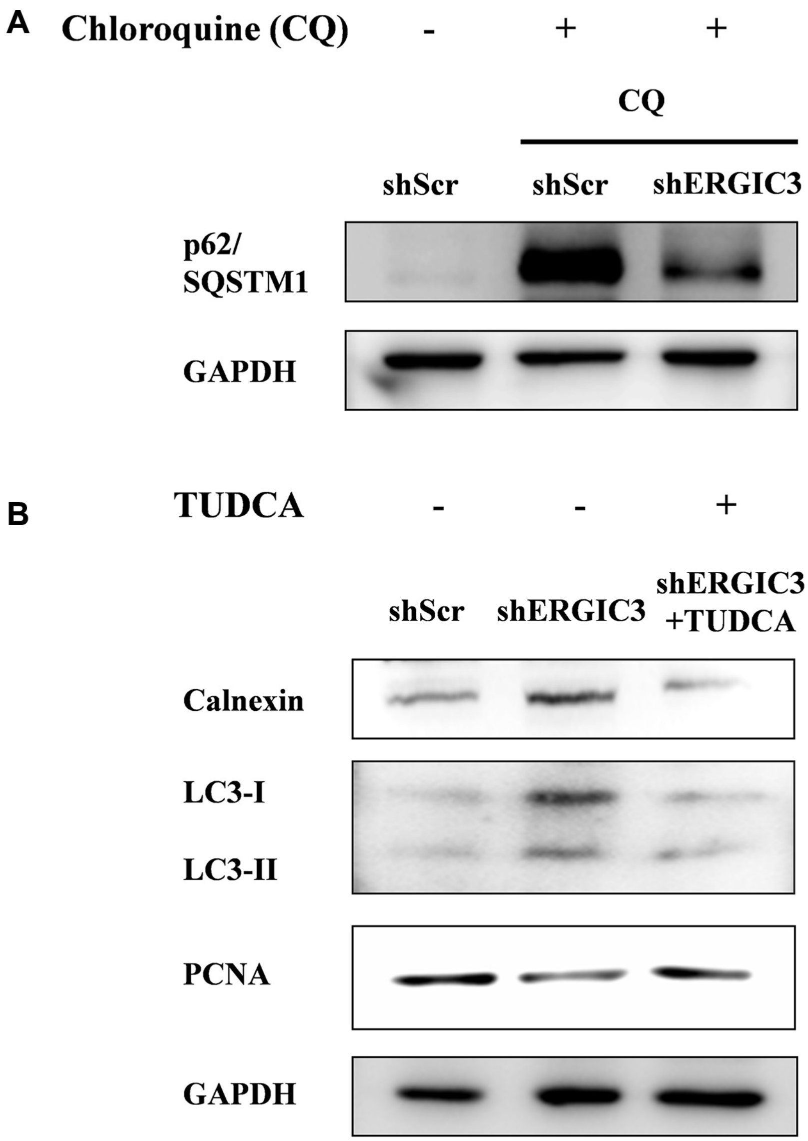

Supplementary Figure S5: Confirmation of roles of ERGIC3 in autophagy and ER stress using chloroquine (CQ) and tauroursodeoxycholic acid (TUDCA). (**A**) Cells were treated with CQ (100 µM) for 24 h in shScr and shERGIC3 stable cell line. (**B**) ERGIC3 downregulated stable cell line (shERGIC3) were cultured for 48 h in the presence or absence of TUDCA (5 mg/mL). shScr, small hairpin scramble; shERGIC3, small hairpin ERGIC3 stable cell line.

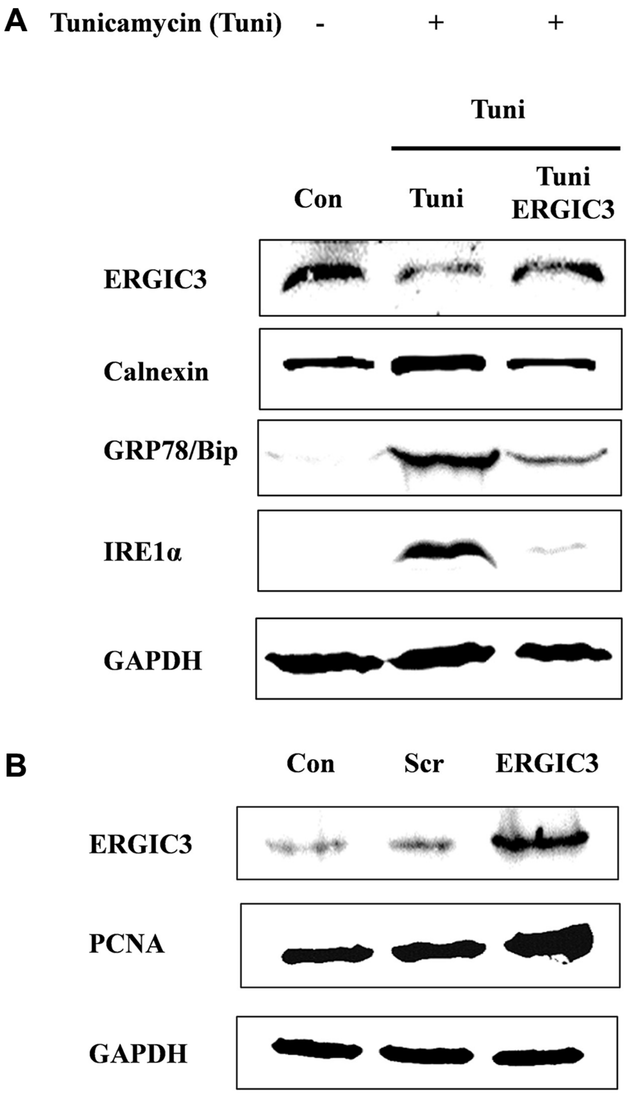

Supplementary Figure S6: Over-expression of ERGIC3 alleviates ER stress and promotes cell proliferation. (**A**) ERGIC3 was transfected into A549 cells and incubated for 24 h, followed by treatment with tunicamycin (5 μg/mL) for 24 h. Western blot assay was performed using ERGIC3, IRE1α, CHOP or Calnexin antibodies. Con, control group; Tuni, tunicamycin treated group; ERGIC3, tunicamycin treated on ERGIC3 over-expressed cell group. (**B**) ERGIC3 was transfected into A549 cells and incubated for 48 h. Western blot assay was performed using PCNA antibody. Con, control group; Scr: scramble control group; ERGIC3: ERGIC3 over-expressed cell group.

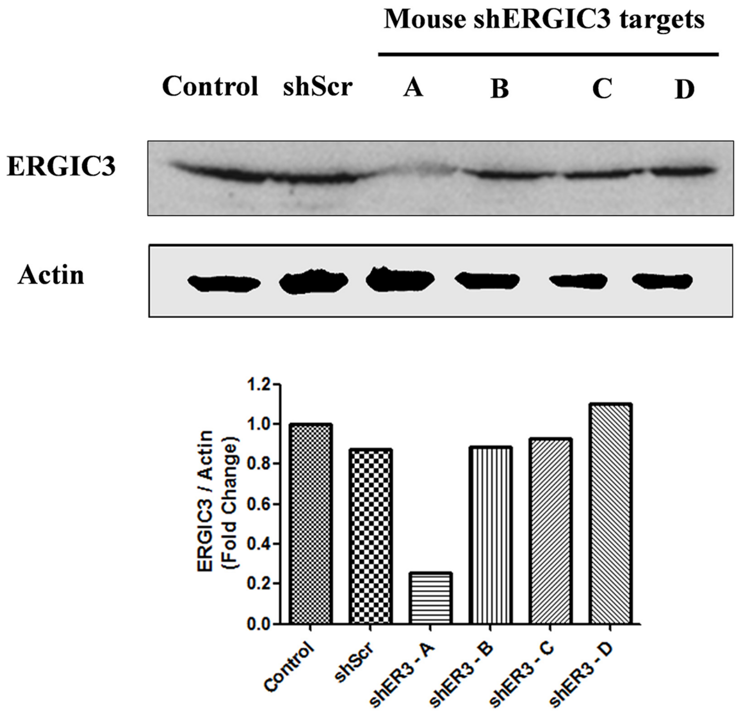

Supplementary Figure S7: Effect of mouse small hairpin ERGIC3 in LA-4 cell line. Western blot was performed after transfection for 48h in LA-4 cells. Target A, ATCGGAGTTGCAGTATTATCTCACTACGG; Target B, GGACACCATTGAGCAG TGTCGGCGAGAGG; Target C, GGAAGAGCTTCCAACAGTCTCATGTGCAT; Target D, TCACCAATGATGGTGAAGCTGAC GGAGAA. Densitometric analysis was performed for the analysis of the ratio ERGIC3 to actin. shScr, small hairpin RNA scramble; shER3, small hairpin RNA of ERGIC3.

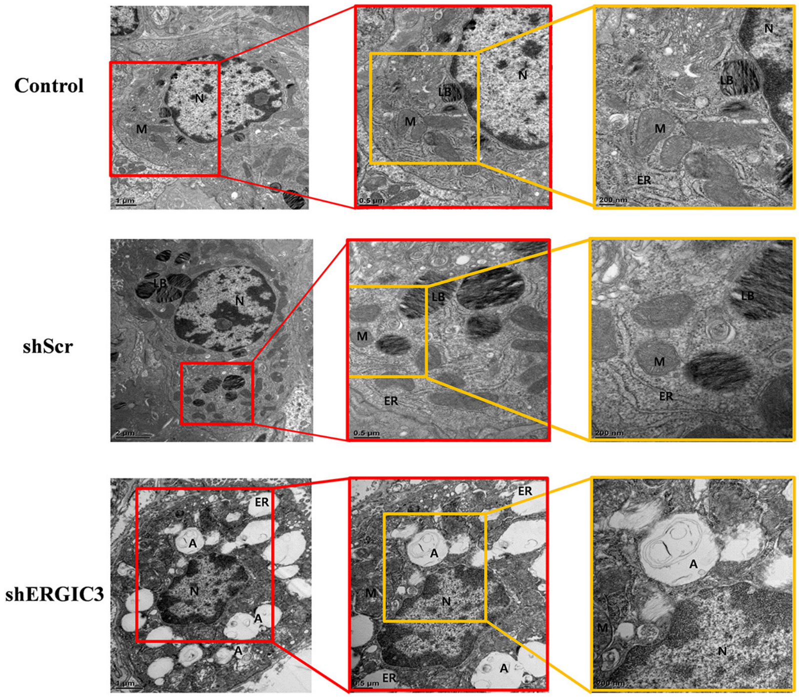

Supplementary Figure S8: Transmission electron microscopy images of lungs in control, shScr and shERGIC3-delivered groups. In the left figures of control and shERGIC3, calibration bars = 1 μm. In the left figure of shScr, calibration bars = 2 μm. In the middle figures of each group, calibration bars = 0.5 μm. In the right figures of each group, calibration bars = 200 nm. N, nucleus; ER, endoplasmic reticulum; M, mitochondria; A, autophagosome; LB, lamellar body. shScr, small hairpin scramble-delivered lung; shERGIC3, small hairpin ERGIC3-delivered lung.

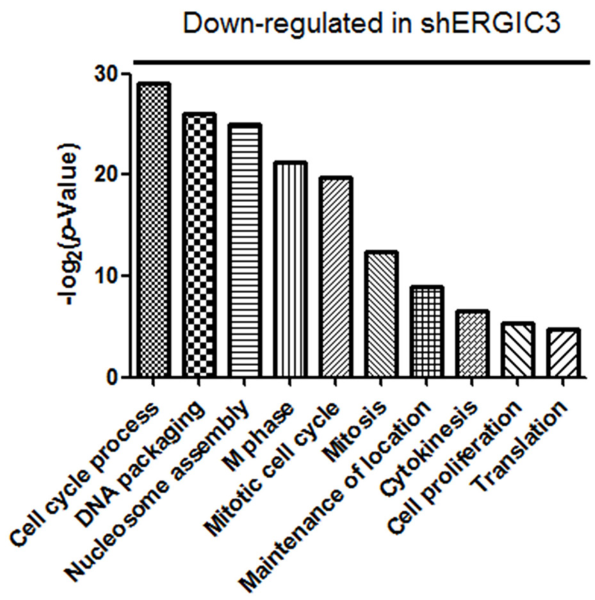

Supplementary Figure S9: Gene ontology analysis for the profiling of important biological processes in shERGIC3. Gene ontology analysis of differentially expressed proteins (DEPs) was performed using DAVID software in which the output is significantly related to biological process in the shERGIC3 down-regulated cell-line.

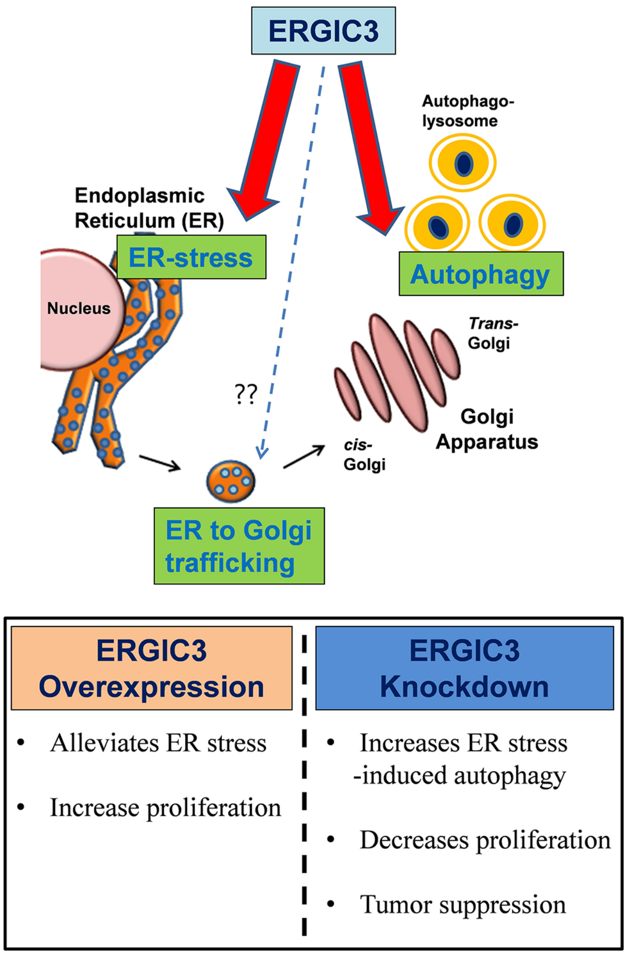

Supplementary Figure S10: A scheme describing the role of ERGIC3 in lung cancer cell.

Yeast two-hybrid screening assay

Matchmaker Gold Yeast Two-Hybrid System (Clontech) was used following the manufacturer's instructions. In brief, ERGIC3 cDNA was cloned in pGBKT7 vector for fusion to the bait Gal4 DNA-binding domain (BD), and prey Mate &Plate Libraries (Clontech, universal human and mouse libraries) were used for expression of Gal4 activation domain (AD). After transformation of the pGBKT7-ERGIC3 construct into yeast cells, mating of bait culture and libraries was performed in synthetic defined (SD) medium/-Trp liquid medium at 30°C for 16 h. For complete library screening, mated cultures were plated on SD/-Leu/-Trp agar plates and incubated for 5 days at 30°C. Positive colonies were inoculated on SD/-Ade/-His/-Leu/-Trp/X-α-Gal/Aureobasidin A plates using yellow pipette tips, and incubated again for 5 days at 30°C. Positive colonies were picked and isolated using Easy Yeast Plasmid Isolation Kit (Clontech).

Sample preparation for proteomics screening

Each sample was digested using modified Filtered Aided Sample Preparation (FASP) according to a previous method [1]. The equal molar of labeled peptides was pooled and fractionated using 3100 OFFGEL fractionator in an immobilized pH gradient gel (12 cm IPG strip pH 3-9, Agilent Technologies Inc., USA) [2]. Samples in each fraction were collected and desalted using a spin column (Harvard Apparatus, USA).

Liquid chromatography-tandem mass spectrometry (LC-MS/MS) analysis

Peptide samples were analyzed using a Q Exactive™ mass spectrometer (Thermo) coupled to an Easy-nLC UPLC system (Easy-nLC 1000; Thermo Fisher Scientific Inc.) and EASY-Spray column (C18, 2 µm particle size, 100Å pore size, 75µm internal diameter × 50cm length, Thermo) as described previously [3].

Protein identification and quantification

All tandem mass spectra were searched against the Universal Protein Resource human protein database (http://www.uniprot.org/) using SEQUEST® data analysis software on Proteome Discoverer 1.4 (Thermo). Tolerance was set to 10 ppm for precursor ions and 0.8 Da for fragment ions. Only fully tryptic ends were considered. Fixed modification options were used for carbamidomethylation (+57.021460 Da) on cysteine, and TMT tag (+229.162932 Da) on lysine and N-terminus. Methionine oxidation (+15.994920 Da) was set as a variable modification. Validation of assigned peptides was based on 1% *q*-values generated by Percolator, a machine-learning supplement to the SEQUEST search algorithm that extends the specificity and sensitivity of peptide identification [4]. Reporter ion intensities from tandem MS spectra were extracted using the Proteome Discoverer software.

Gene ontology (GO) analysis to profile biological processes in shERGIC3 cell-line

To unravel proteome change and subsequent alterations in biological functions in the shERGIC3 condition, proteome quantitative analysis using tandem mass tag (TMT) labelling was performed. Using the Isobar (R software package), protein ratios, ratio *P*-value and sample *P*-value for each quantitated protein estimating technical variability were calculated. Through a comparative study of control and shERGIC3 samples, we discovered differentially expressed proteins (DEPs) whose sample and ratio *P*-values were below 0.05 in at least one replicate. Using those DEPs, GO analysis was performed to in order to reveal enriched GO of significantly changed proteins. Using the DAVID bioinformatics resource program, we selected the significantly enriched GO annotation from DEPs when they had a P value < 0.05 in Fisher exact test. Some biological process terms were enriched that are presented as a graph in Supplementary Figure S9. GO analysis of DEPs revealed that biological processes related to cell cycle, DNA packaging, nucleosome assembly, M-phase, mitotic cell cycle, mitosis, maintenance of location, cytokines, cell proliferation and translation were representative of down-regulated proteins in shERGIC3.

REFERENCES

1. Kim SJ, Chae S, Kim H, Mun DG, Back S, Choi HY, Park KS, Hwang D, Choi SH, Lee SW. A protein profile of visceral adipose tissues linked to early pathogenesis of type 2 diabetes mellitus. Mol Cell Proteomics. 2014; 13:811–822.
2. Horth P, Miller CA, Preckel T, Wenz C. Efficient fractionation and improved protein identification by peptide OFFGEL electrophoresis. Mol Cell Proteomics. 2006; 5:1968–1974.
3. Choi DY, You S, Jung JH, Lee JC, Rho JK, Lee KY, Freeman MR, Kim KP, Kim J. Extracellular vesicles shed from gefitinib-resistant nonsmall cell lung cancer regulate the tumor microenvironment. Proteomics. 2014; 14:1845–1856.
4. Käll L, Canterbury JD, Weston J, Noble WS, MacCoss MJ. Semi-supervised learning for peptide identification from shotgun proteomics datasets. Nat methods. 2007; 4: 923–925.

Supplementary Table S1: Yeast two hybrid results of ERGIC3

Symbol	Full name	Subcellular localization
IER3IP1	Immediate early response 3 interacting protein 1	ER, Golgi apparatus
SNAPIN	SNAP-associated protein	Golgi apparatus, Cytosol, Nucleus
RYR2	Ryanodine receptor 2 (cardiac)	ER, Plasma membrane
DPP4	Depeptidyl-peptidase 4	Plasma membrane, ER, Golgi apparatus
RRBP1	Ribosome binding protein 1	ER
CLN5	Ceroid-lipofuscinosis, neuronal 5	ER, Golgi apparatus, Lysosome
RER1	Retention in endoplasmic reticulum sorting receptor 1	ER, Golgi apparatus, Plasma membrane
TPT1	Tumor protein, translationally-controlled 1	Endosome, Cytosol, Nucleus
IPO7	Importin 7	Nucleus, Golgi apparatus, Cytosol
EIF3H	Eukaryotic translation initiation factor 3, subunit H	Cytosol , Nucleus, ER, Mitochondria
WWC2	WW and C2 domain containing 2	Cytosol
SKAP2	Scr kinase associated phosphoprotein 2	Plasma membrane, Cytosol, Nucleus
FAF1	FAS-associated factor 1	Plasma membrane, Cytosol, Nucleus
SESN3	Sestrin 3	Mitochondria, Cytosol, Nucleus
ARG1	Arginase 1	Mitochondria, Cytosol, Nucleus
CDK2AP1	Cyclin-dependent kinase 2-associated protein 1	Mitochondria, Cytosol, Nucleus
OPN1SW	Opsin 1 (cone pigments), short-wave-sensitive	Plasma membrane
HTR1E	5-hydroxytryptamine (serotonin) receptor 1E, G protein-coupled	Plasma membrane
RAB18	RAB18, Member RAS oncogene family	Plasma membrane, Cytosol, Nucleus, Golgi apparatus, Mitochondria
HSPA4	Heat shock 70 kDa protein 4	Nucleus, Cytoskeleton, Cytosol, ER, mitochondria, Golgi apparatus
RDX	Radixin	Cytoskeleton, Plasma membrane, Cytosol, Golgi apparatus, Nucleus
PEA15	Phosphoprotein enriched in astrocytes 15	Cytoskeleton, Cytosol
ARL2BP	ADP-ribosylation factor-like 2 binding protein	Cytoskeleton, Mitochondria, Nucleus
LCE6A	Late cornified envelope 6A	Cytosol, Nucleus
ANP32C	Acidic (leucine-rich) nuclear phosphoprotein 32 family, member C	Cytosol, Nucleus
SNRPG	Small nuclear ribonucleoprotein polypeptide G	Cytosol, Nucleus
ARL15	ADP-ribosylation factor-like 15	Cytosol, Nucleus
CENPP	Centromere protein P	Cytosol, Nucleus
RICTOR	RPTOR independent companion of mTOR, complex 2	Cytosol, Nucleus
MAP3K1	Mitogen-activated protein kinase kinase kinase 1	Cytosol, Nucleus
KRCC1	Lysine-rich coiled-coil 1	Cytosol, Nucleus
KDM1B	Lysine-specific demethylase 1B	Cytosol, Nucleus
RFN10	Ring finger protein 10	Cytosol, Nucleus
GGNBP2	Gametogenetin-binding protein 2	Cytosol, Nucleus
LRRC58	Leucine rich repeat containing 58	Cytosol, Nucleus

We referred localization information of each protein from GeneCards website (http://www.genecards.org/).
